# Isolating the Vibrational Spectra of the Red Chlorophylls in Photosystem I with Multispectral Two-Dimensional Spectroscopy

**DOI:** 10.1021/acs.jpclett.6c00658

**Published:** 2026-05-12

**Authors:** James D. Shipp, Chenshuai Li, Yumin Lee, Michael Gorka, John H. Golbeck, Jessica M. Anna

**Affiliations:** † Department of Chemistry, 6614 University of Pittsburgh, Chevron Science Center, 219 Parkman Avenue, Pittsburgh, Pennsylvania 15260, United States; ‡ Department of Chemistry, University of Pennsylvania, 213 S. 34th Street, Philadelphia, Pennsylvania 19104, United States; § Department of Chemistry and Chemical Biology and The Baruch ’60 Center for Biochemical Solar Energy Research, Rensselaer Polytechnic Institute, Troy, New York 12180, United States; ∥ Department of Biochemistry and Molecular Biology, The Pennsylvania State University, University Park, Pennsylvania 16802, United States

## Abstract

Photosystem I (PSI) uses an antenna of chlorophyll (Chl) molecules to create a charge separated state with high quantum efficiency. Understanding the charge separation mechanism is currently hindered by spectral overlap between the antenna and reaction center (RC) Chls and the fact that energy transfer and electron transfer occur with similar time scales. Here, we characterize the antenna excited states by applying two-dimensional electronic (2DES) and two-dimensional electronic-vibrational (2DEV) spectroscopy to PSI complexes with closed RCs. Comparison of the 2DES and 2DEV spectra, which evolve with the same kinetics, enabled characterization of the vibrational modes of the antenna during energy equilibration between spectrally distinct Chls. Through global analysis, we learn how energy transfer between the Bulk and Red Chls presents in the 2DEV spectra and we definitively identify vibrations of the cationic components of the mixed exciton and intermolecular charge transfer states associated with the Red Chls. This work enables future studies of the initial charge separation mechanism of PSI by 2DEV spectroscopy.

## Graphical abstract

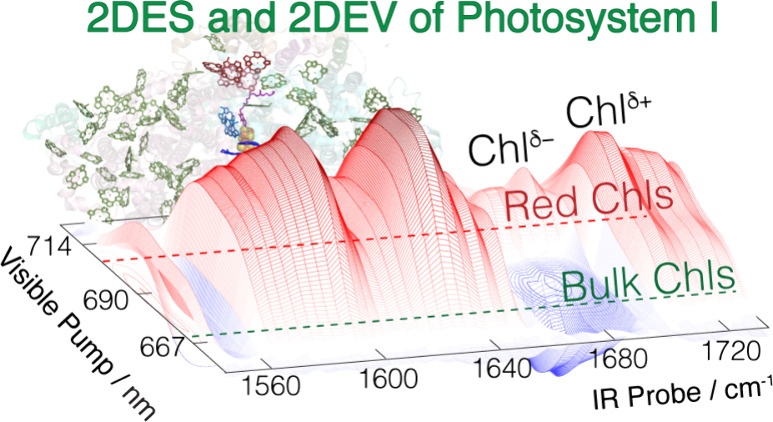

Photosystem I (PSI) drives oxygenic photosynthesis by harvesting sunlight and performing highly efficient charge separation (CS). *In vivo*, CS results in electron transfer to a soluble ferredoxin, where the energy is ultimately used in carbon fixation. When isolated, the charge separated state (CSS) is long-lived and can be harnessed in photobioelectrochemical cells.^1,2^ To achieve efficient light capture and CS, PSI uses a network of Chl molecules arranged into an antenna that absorbs photons and funnels the excitation energy to the Reaction Center (RC) core, a cluster of six Chls wherein CS takes place with nearly 100% quantum efficiency.^3–6^ The structure of PSI isolated from *Synechocystis* sp. PCC 6803 (PDB: 5OY0),^7^ which we study in this manuscript, is shown in monomeric form in Figure 1.^7^ Each PSI monomer contains 95 Chls. Of the six Chls in the RC core, the special pair (P_700_) is the lowest energy site. The remaining 89 Chls comprise the light harvesting antenna. The majority of antenna Chls absorb light at higher energies relative to P_700_ and are known as the ‘Bulk Chls’ (green Chls, Figure 1), where their absorption wavelengths are tuned by the local protein environment. A few of the antenna Chls form dimers and timers that have electronic transitions that lie at lower energies compared to P_700_, and these are known as the ‘Red Chls’.^8–15^ The location of the Red Chls within the cyanobacterial PSI antenna are species dependent; in *Synechocystis* sp. PCC 6803 the dimeric and trimeric Red Chl sites have been identified and are shown in red in Figure 1.^14^ The red-shifted transitions of the Red Chls result from the tuning of the absorption wavelength by the local environment and the spatial arrangement of the Chls (Figure 1). The close proximity of the Chls within a Red site leads to the formation of Frenkel excitonic (FE) states, which are linear combinations of the local excited states (Chl*Chl and ChlChl* for a dimer), In addition, molecular orbital overlap can lead to the formation of interchlorophyll charge transfer states (Chl^–^Chl^+^ and Chl^+^Chl^–^ for a dimer). The mixing of the local excited states with the CT states results in mixed exciton-CT states, where the mixing also contributes to the red shift of the Red Chl states, and the lowest energy mixed state has both FE and CT character.^16,17^ Previous studies have focused on characterizing the mixed FE/CT states of the Red Chls of PSI complexes isolated from plants^17–20^ and cyanobacteria^16,21^ using a range of techniques including transient absorption,^22,23^ fluorescence,^14,24,25^ hole-burning,^21,26–28^ and Stark spectroscopies.^18,19,29–31^

**Figure 1:**
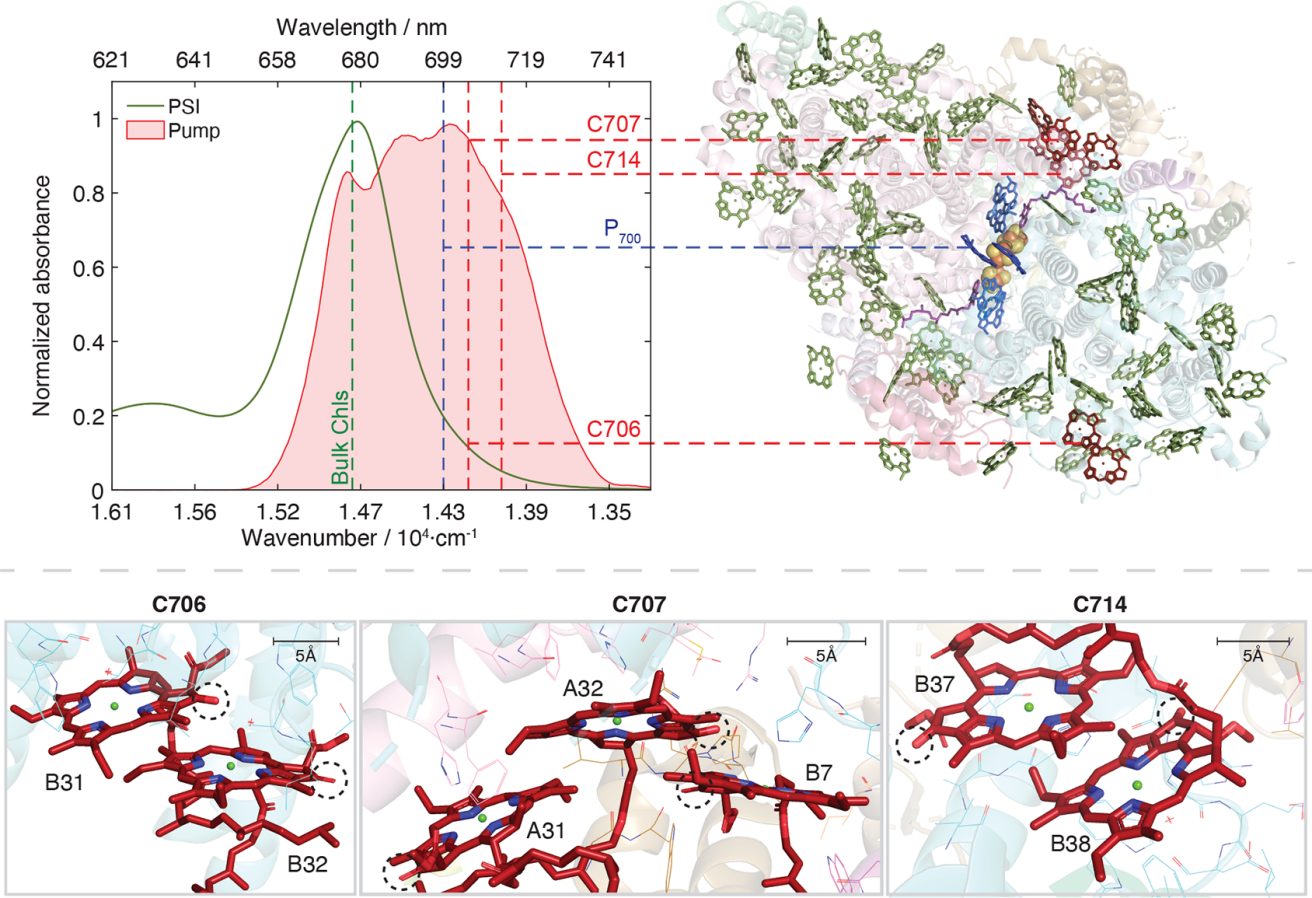
Normalized UV–vis spectrum of PSI extracted from *Synechocystis* sp. PCC 6803 in 50 mM D_2_O Tris buffer (pH 8.3) solution; the absorption wavelengths of the Bulk Chl Q_*y*_ (green), P_700_ (blue), and Red Chls (red) are highlighted, alongside the relevant sites in the monomeric structure of PSI (PDB: 5OY0). The spectrum of the visible pump pulses is shown in the red shaded area. The Red Chls are labeled according to previous work, where the absorptions at 706, 707, and 714 nm have been assigned to the B31-B32, B7-A31-A32, and B37-B38 Chls, respectively.^14^ The local environment of each Red Chl site is also shown, including nearby amino acid residues within 5 Å of the Chls. The 13^1^-keto carbonyl groups that are used as a vibrational probe are highlighted with dashed circles.

At present, we have a general understanding of how energy flows between the spectrally distinct antenna Chls to reach the RC core, and how the CSS evolves on longer time scales.^8,9,28,32–37^ However, there are fundamental aspects of the initial stages of CS in the RC core that remain unknown,^34^ including the identity of the primary electron donor.^38^ Answers to these mechanistic questions are key aspects to developing our understanding of the high quantum efficiency in PSI. However, characterization of the initial mechanism of CS in PSI is difficult, primarily due to the severe spectral and temporal congestion in the spectroscopic data. Unlike PSII, in PSI the RC core cannot be isolated from the antenna Chls. Thus, the smallest isolated PSI complexes contain RC Chls, Red Chls, and Bulk Chls, with overlapped Q_*y*_ absorption bands at room temperature. In addition to spectral congestion, PSI complexes also exhibit temporal congestion where the antenna excited states evolve on similar time scales (fs to ps) to the initial CS processes in the RC core. Therefore, the Q_*y*_ absorptions associated with the CSS in the RC core will always be convolved with the spectral features of the Bulk and Red Chls.

Ultrafast time-resolved infrared (TRIR) spectroscopy has been shown to be a powerful technique for characterizing CS in photosynthetic systems.^39^ The vibrational absorption bands of the Chls have much narrower line widths than the electronic bands and are also sensitive to the electron density of the Chls and their local electrostatic environment. Previous studies on PSI by Di Donato et al.^40^ and van Thor et al.^41^ have effectively demonstrated the utility of TRIR for characterization of energy transfer and CS in PSI. The TRIR experiments revealed absorption bands of cationic Chls that form on ultrafast time scales. However, in these studies, the cationic Chl features were not definitively assigned to a particular Chl, and were ascribed to either the Red Chl charge transfer states or to CS at the RC core on sub-picosecond time scales.^40,41^ Another possibility is the previously observed Chl cationic feature had overlapping contributions from both the Red Chls and the RC core.

To study initial CS in the RC core, a definitive assignment of the Chl cationic features of the Red Chl and RC excited states is required. In this work, we focus on characterization of the Red Chl excited states in PSI complexes. To resolve the vibrational absorption spectrum of the Red Chls, we use multispectral two-dimensional (2D) spectroscopies in the visible and mid-IR regions.

2D spectroscopy provides both high spectral and temporal resolution and is therefore an excellent technique for the investigation of photosynthetic systems.^10,42–47^ For example, 2DES has been used to characterize energy transfer in light harvesting complexes.^48^ In a 2DES experiment, two broadband visible pump pulses, separated by the t_1_ time delay, excite the antenna Chls. The evolution of the Chl excited states is then tracked over time by a visible probe pulse separated from the pump pulses by the population time delay (t_2_). Fourier transformation of the detected signal along the t_1_ axis produces a 2D correlation map, where peaks along the diagonal line represent the self-responses of the excited states, and ‘cross-peaks’ in off-diagonal positions report on energy transfer between spectrally distinct Chl sites. 2DEV has also been used to characterize energy transfer and CS in light harvesting complexes.^44,45,49–51^ In a 2DEV experiment,^49^ the visible probe is replaced with a broadband mid-IR pulse to detect cross-correlations between the electronic states and vibrational modes of the Chls. In effect, in 2DEV spectra, cross-peaks are observed between the electronic excited states and vibrational normal modes.^52^ Recently, the Ogilvie group has demonstrated multispectral 2D spectroscopy, where the combination of 2DES and 2DEV spectroscopy was highly effective for characterization of the CS mechanism in PSII.^44^ The combination of 2DES and 2DEV is powerful as the spectroscopic features of an excited state in the two probed regions evolve with the same kinetics.^44^

2DES has also been used to study energy transfer in PSI,^36,42,53^ but 2DEV measurements have not yet been performed on PSI. We seek to use 2DEV to characterize the mechanisms of CS in the PSI RC core, as has been demonstrated for PSII.^44^ However, as noted above, the PSI RC core cannot be isolated from the antenna Chls, hence we must first understand how to interpret the signals associated with the Bulk and Red Chls in the 2DEV spectra as well as how they evolve over time. To achieve this, we apply both 2DES and 2DEV spectroscopy to cyanobacterial PSI trimers from *Synechocystis* sp. PCC 6803. To isolate the spectral response of the antenna, we perform our measurements on closed reaction centers, in which the primary donor, P_700_, is in the oxidized state, thereby enabling characterization of the initial antenna excited states without overlapping contributions from the charge separation processes occurring within the RC. Our measurements were performed at room temperature to enable uphill energy transfer from Red to Bulk Chls and we use the well characterized equilibration kinetics in the 2DES spectra to interpret the vibrational absorption features in the 1660–1730 cm^–1^ spectral region. Through 2D global fitting, we definitively assign the photoinduced vibrational absorption at ∼ 1708 cm^–1^ in the 2DEV spectra to Chl cationic features (Chl^δ+^) of the mixed exciton-CT states of the Red Chls. This work, which presents the first 2DEV spectra of PSI, lays a foundation for future characterization of the early stages of charge separation within the PSI RC with multispectral multidimensional spectroscopic techniques.

Multispectral 2D spectroscopy was performed on PSI complexes with photo-oxidized RCs, where CS is not possible due to the presence of oxidized P_700_ (See SI, Section S2). The prevention of CS in PSI allows us to focus on the energy transfer processes within the antenna. The spectra of the incoming visible laser pulses are plotted in Figure 1 (See SI, Figure S1 for mid-IR pulse spectrum) and the multispectral data are presented in Figure 2A, which show the response of the PSI antenna in the visible and mid-IR regions following electronic excitation. The 2D spectra contain overlapped positive (red) and negative (blue) peaks. In the 2DES, positive features are assigned to electronic excited state absorption (ESA) bands of the Chls. The negative bands are attributed to Chl ground state bleach (GSB) and stimulated emission (SE) transitions. The initial dominant features of the 2DES spectra (Figure 2A, Top) are the GSB/SE signals of the Red Chl (above 700 nm) or Bulk Chl (below 700 nm). Due to the spectral congestion, we cannot fully resolve cross-peaks in the 2DES spectra. However, as the t_2_ delay time increases, off-diagonal growths are evidenced through apparent changes in the spectral line shape. These features grow in due to energy transfer between the Bulk and Red Chls. On longer time scales, the GSB/SE bands decay toward the baseline as energy is trapped by the RC core.

**Figure 2:**
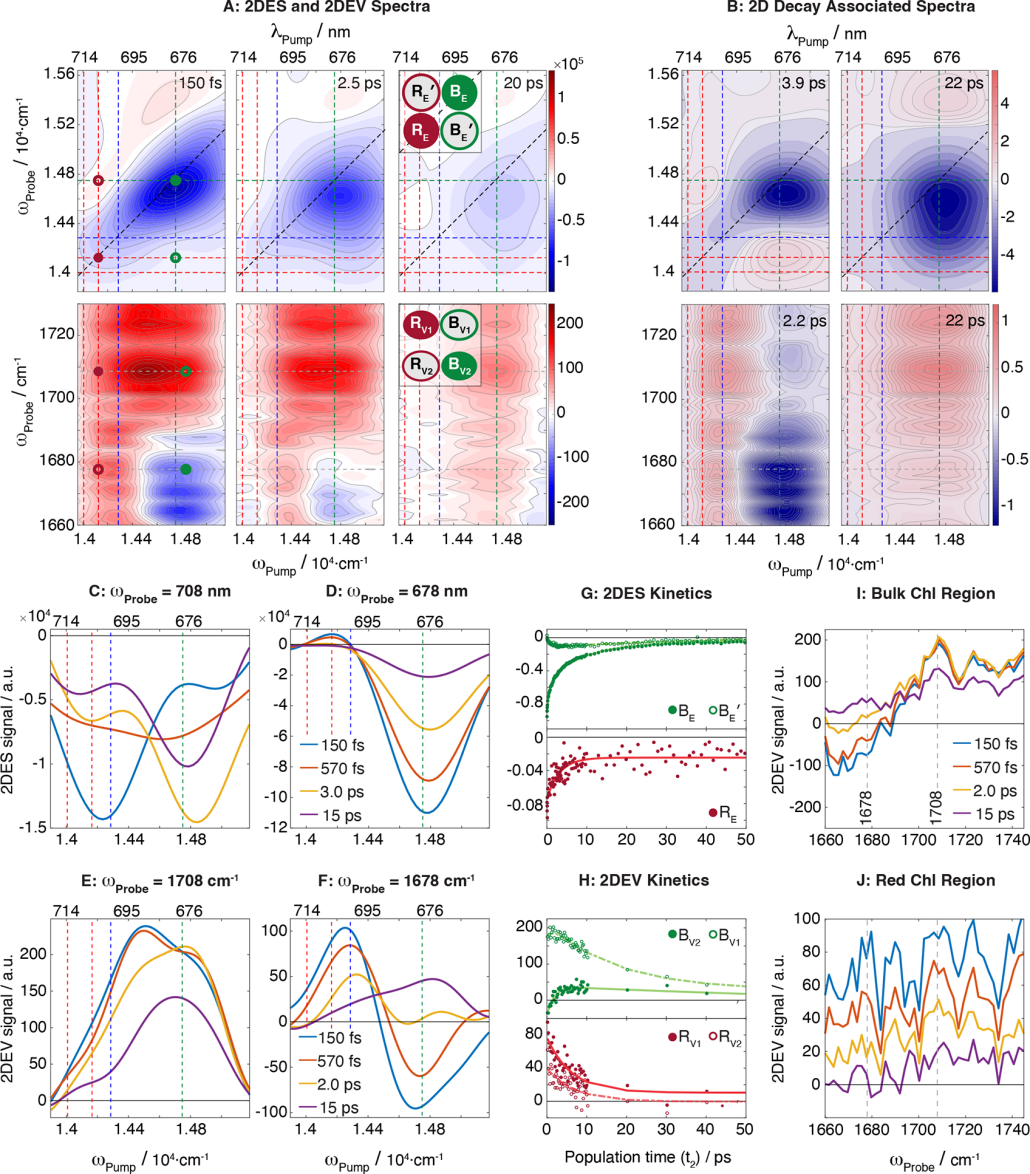
(A) 2DES and 2DEV spectra of PSI extracted from *Synechocystis* sp. PCC 6803 in Tris buffer pH 8.3, collected following excitation with a visible pulse spanning the 660–740 nm (1.35 × 10^4^–1.52 × 10^4^ cm^–1^) spectral region and detection with a visible pulse spanning the 620–746 nm (1.34 × 10^4^– 1.61 × 10^4^ cm^–1^) region or mid-IR pulse spanning the 1660–1740 cm^–1^spectral region. Correlation lines are drawn at pump wavelengths of 714, 708, 700, and 678 nm (1.400 × 10^4^, 1.412 × 10^4^, 1.43 × 10^4^, and 1.475 × 10^4^ cm^–1^). (B) 2D-DAS obtained from the multispectral data, where positive values (red) correspond to positive peak decay or negative peak growth and negative values (blue) correspond to positive peak growth or negative peak decay. (C–F) Slices taken along the pump axis at the detection axis positions given above each plot. The t_2_ delay at which each spectrum is obtained is displayed in panels D and F, respectively. (G, H) Kinetic traces obtained from excitation of the Bulk and Red Chls in the 2DES and 2DEV experiments taken at frequency coordinates represented by the scheme in the inset of (A). (I, J) Representative slices along the probe axis taken from the 2DEV spectra for excitation of the Bulk and Red Chls at the t_2_ delays shown in panel (I).

The 2DEV spectra (Figure 2A, Bottom), report on the same excited state dynamics as the 2DES and are comprised of many overlapping positive and negative vibrational transitions. In 2DEV spectra, the positive bands correspond to vibrations of the excited electronic states, while the negative bands correspond to vibrations of the ground electronic states. When analyzing the 2DEV spectra we will focus on absorptions of the Chl 13^1^-keto carbonyl (C=O) groups. Previous TRIR studies,^40,41^ have assigned transitions of the 13^1^-keto groups of both ground and excited state Chls and demonstrated that these transitions are sensitive to the charge of the Chls: neutral Chls in the electronic ground state (GS) and electronic excited state (ES) have vibrations in the 1640–1670 cm^–1^ and 1610–1650 cm^–1^ regions, respectively, while cationic Chls have 13^1^-keto vibrations between 1700–1730 cm^–1^.^38,40,41,54,55^ 2DEV spectra spanning the 1545–1800 cm^–1^ region are reported in the SI, where 13^1^-keto transitions of the neutral GS, neutral ES, and cationic Chl bands are observed. In this work, we focus on the vibrational band at 1708 cm^–1^ that has been previously attributed to the Chl cation features of either the Red Chl states or the initial CS in the RC core; where the peak corresponds to a 13^1^-keto mode of Chls with reduced electron density (Chl^δ+^). For analysis of the 1708 cm^–1^ band we focus on 2DEV spectra spanning the 1660–1740 cm^–1^ region (Figure 2A) recorded at a higher detection axis resolution, albeit with a smaller spectral range. In this region we primarily monitor the neutral Chls in the GS and the Chl cation band. We will use these vibrational modes as reporters to identify changes in the Chl electron densities during energy transfer between Bulk and Red Chls.

Multispectral 2D spectroscopy frequency resolves the response of the Bulk and Red Chls along the excitation axis. To highlight the dependence of the spectral response on the excitation frequency, slices along the ω_Pump_ axis were taken at specific ω_Probe_ frequencies (Figure 2C–F). For the 2DES data, slices along the pump axis were taken at probe wavelengths of 678 nm (1.47 × 10^4^ cm^–1^) and 708 nm (1.41 × 10^4^ cm^–1^), which are associated with the Bulk or Red Chls, respectively (Figure 2C,D). At short time delays, the strongest signal is found at the diagonal coordinate, where ω_Pump_ = ω_Probe_. As the delay time increases, these diagonal peaks decay and a growth in the cross-peak region occurs – a marker of energy transfer between the Bulk and Red Chls.^36,42,43^

The change in amplitude of the 2DES features with increasing t_2_ delay is shown in Figure 2G, where the kinetic traces are obtained from the points marked in the 2DES spectra in Figure 2A and noted by the schematic spectra (Figure 2A, Inset). The transitions are noted in the schematic spectra where peaks R_E_ and B_E_ are the Red and Bulk Chl diagonal peaks, which are at their highest intensity at t_2_ ≈ 0 ps and decay toward the baseline on the picosecond time scale. Peaks R_E_′ and B_E_′ denote where cross-peaks would appear due to equilibration between the Bulk and Red Chls. Peak R_E_′ arises from excitation of the Red Chls and detection of the Bulk Chls whereas peak B_E_′ arises from excitation of the Bulk Chls and detection of the Red Chls. In the experimental spectra, clear cross-peaks are not observed due to spectral congestion, but we can still monitor equilibration as a growth in the cross-peak region.^42^ Here we focus on analysis of the B_E_′ cross-peak region rather than the R_E_′ cross-peak region, as the B_E_′ cross-peak region lies below the diagonal and thus has minimal overlap with the ESA features. The B_E_′ cross-peak region has a very small initial amplitude and grows in on the picosecond time scale, reaching the maximum amplitude approximately 3 ps after excitation. After the growth is complete, Peak B_E_′ decays to the baseline as energy is quenched by P_700_^+^. The kinetic traces and slices obtained illustrate how the equilibration process manifests in the 2DES spectra, similar to previous studies on PSI.^36,42,43^

The response of the 13^1^-keto vibrations in the 2DEV spectra were also found to change with excitation frequency, as highlighted by slices taken along the pump axis at mid-IR detection frequencies of 1678 cm^–1^, where we see a negative transition associated with the Bulk Chls, and 1708 cm^–1^, which has been assigned to a Chl cation band in the previous TRIR studies^40,41^ (Figure 2E,F). In the early t_2_ time slices at ω_Probe_ = 1678 cm^–1^, positive peaks are observed after excitation of the Red Chls, while negative features are found at higher pump energies after excitation of the Bulk Chls. As the t_2_ time increases, all the spectral features become positive, as shown by the relevant kinetic traces in Figure 2H. In the slice along the pump axis at ω_Probe_ = 1708 cm^–1^ we observe positive features spanning the Bulk and Red Chl regions. At early t_2_ times we observe a maximum intensity at 689 nm (1.45 × 10^4^ cm^–1^), as the t_2_ delay increases the amplitude at 689 nm rapidly decays and at later t_2_ times the amplitude is distributed between the Red and Bulk Chls. We note that the intensity profile of the slices along the pump axis can be impacted by the spectrum of the incoming pump pulses (Figure 1 and Figure S7 in SI Section S7) as different groups of Chls could be preferentially excited.

To further characterize the vibrational response to excitation of the Bulk or Red Chls, slices were taken along the ω_Probe_ axis at ω_Pump_ = 708 nm (1.41 × 10^4^ cm^–1^) corresponding to the Red Chls, and at ω_Pump_ = 674 nm (1.484 × 10^4^ cm^–1^) corresponding to the Bulk Chl region (Figure 2I,J, see Figure S4 for additional slices along the mid-IR probe axis across a broader spectral range). The spectral features at 1676 and 1708 cm^–1^ are marked by the dashed lines superposed on the slices and are assigned in the following sections on the basis of previous FTIR and TRIR studies performed on PSI,^40,41,54,56–58^ as well as electronic structure calculations of Chl a.^59^

Excitation of the Bulk Chls, monitored at the blue edge of the spectrum at 674 nm, results in slices along the 2DEV probe axis that closely resemble the reported TRIR spectra of PSI.^40,41^ A prominent negative feature was observed between 1661–1695 cm^–1^, attributed to the GS 13^1^-keto modes. The kinetic traces in Figure 2H show that the negative features (B_V2_) are at the maximum negative amplitude at the earliest measured t_2_ delay (t_2_ = 150 fs). These peaks then decay toward the baseline concomitant with new positive features emerging at ≈1678 cm^–1^, causing the signal amplitude to become increasingly positive over time as the negative peaks decay. Multiple overlapping peaks were also observed in the 1700–1730 cm^–1^ region. Here we focus on the peak at ω_Pump_ = 1.484 × 10^4^ cm^–1^ (674 nm) ω_Probe_ = 1708 cm^–1^ (B_V1_), a signal associated with Chl^δ+^. The slices along the probe axis at ω_Pump_ = 1.484 × 10^4^ cm^–1^ and kinetic traces in Figure 2 show an initial positive amplitude at 1708 cm^–1^. Then, the peak at 1708 cm^–1^ undergoes a growth on the picosecond time scale, followed by a decay. Additional traces from 2DEV spectra across a broader detection range are plotted in the SI (Section S4) and are consistent with Figure 2.

The 2DEV spectra of PSI after excitation of the Red Chls, monitored at the red edge of the spectra (708 nm, Figure 2J), are different to those obtained at the Bulk Chl excitation. The key change is the presence of intense positive peaks between 1662–1690 cm^–1^. The kinetic trace at ω_Pump_ = 1.412 × 10^4^ cm^–1^ (708 nm) ω_Probe_ = 1678 cm^–1^ (R_V2_ in Figure 2H) shows the signal amplitude in this region is initially at its maximum amplitude and then decays toward the baseline on the picosecond time scale. The positive features overlap with and cancel out the expected negative features associated with the 13^1^-keto vibrations in the GS that are initially observed after Bulk Chl excitation. Therefore, the expected negative features associated with the electronic GS are not observed following Red Chl excitation due to the overlap with these positive peaks. Positive peaks were also observed in the 1700–1730 cm^–1^ region after exciting the Red Chls, and are also assigned to Chl^δ+^ vibrations, consistent with previous TRIR studies.^40,41^ The kinetic trace at ω_Pump_ = 1.412 × 10^4^ cm^–1^ ω_Probe_ = 1708 cm^–1^ (R_V1_ in Figure 2H) shows that the Chl^δ+^ peaks formed after Red Chl excitation decay toward the baseline with no initial growth of the signal intensity, unlike what is observed in the Bulk Chl pump region. We note that as CS does not occur under the experimental conditions used here (See SI, Section S2), the dynamic Chl^δ+^ features do not arise from the pre-oxidized RC core.

The 2DES and 2DEV spectra both probe energy transfer in the PSI antenna, and thus their temporal evolution is governed by the same photoinduced kinetics. Now, we will compare the time-dependent peak amplitudes in the 2DEV spectra with the well-characterized evolution of the electronic GSB/SE peaks (R_E_, B_E_, and B_E_′) in the 2DES to identify vibrational spectroscopic reporters for the Red Chls and determine how equilibration among the antenna Chls presents in the 2DEV spectra.

The positive vibrational absorption feature at 1708 cm^–1^ (R_V1_), corresponding to the 13^1^-keto vibration of Chl^δ+^, decays following Red Chl excitation. Upon Bulk Chl excitation (B_V1_), this feature shows instead an initial growth that maximizes between t_2_ = 2–3 ps, followed by a decay. The same kinetics can be found in the 2DES spectra, where the Red Chl diagonal peak (R_E_) decays, while the downhill energy transfer cross-peak (B_E_′) grows in and then decays. This similarity in the peak kinetics suggests the Chl^δ+^ feature is associated with the Red Chl sites, where the 2DEV spectral evolution is consistent with the 2DES equilibration kinetics of the Bulk and Red Chls.

This comparison can also be applied to the peaks in the 1660–1690 cm^–1^ region (R_V2_, B_V2_). The positive peaks initially observed after Red Chl excitation decay toward the baseline (analogous to peak R_E_), while we see growth of positive features, overlapped with the initial negative peaks, in this region after exciting the Bulk Chls. Consistent with the peaks at 1708 cm^–1^ (V1 peaks) the evolution of these positive vibrational features may also report on the Bulk Chl to Red Chl energy transfer kinetics, hence they can be assigned to the Red Chl sites. These assignments are reinforced by global fitting of the multispectral data, where the 2D-Decay Associated Spectra (2D-DAS) are shown in Figure 2B and analyzed below.

To further confirm the assignment of the vibrational absorption features of the Red Chls, a global analysis was performed to obtain 2D-DAS. Details on the global fitting procedure are provided in the SI, Section S6. The 2DEV and 2DES data were fit independently to ensure that the analysis and interpretation of the results are consistent across experimental techniques. The 2DEV-DAS are shown in Figure 2B, where the two kinetic components evolve on a 2.2 and 22 ps time scale. Two of the 2DES-DAS (SI, Figure S2), which have been characterized previously,^42^ have similar decay constants to the 2DEV-DAS and are also presented in Figure 2B. We note that the IR probe pulse has a larger temporal bandwidth compared to the visible pulses, and thus it was not possible to isolate sub-picosecond time scale components from the 2DEV spectra that were observed in the 2DES-DAS, where the 2DES-DAS 1 and 2DES-DAS 2 were previously assigned to ultrafast relaxation and fs equilibration among unrelaxed Chls.^42^ The similar kinetics allow us to assign the two 2DEV-DAS to the same processes as 2DES-DAS 3 and 4. Thus, the kinetics observed in the 2DEV spectra are attributed to equilibration on picosecond time scale and energy quenching by P_700_^+^. We consider the picosecond processes to occur between vibrationally relaxed excited states. This is consistent with previous measurements that have shown vibrational cooling takes place on the femtosecond time scales for excited Chls in protein environments.^60^ This is also consistent with our previous 2DES studies on PSI, where the picosecond processes were not greatly impacted by the pump pulse tuning.^42^

The equilibration 2D-DAS (2DES-DAS 3 and 2DEV-DAS 1) obtained from the electronic and vibrational probes also have very similar pump frequency dependencies. In the 2DES spectra, if we excite and then detect the Red Chls, we observe decay of their diagonal peaks (R_E_). Conversely, if we excite the Bulk Chls, a Red Chl growth is observed in the lower cross-peak region (B_E_′). Identical behavior is also evident for the positive 13^1^-keto peaks in 2DEV-DAS 1 (R_V1_, B_V1_). Thus, the multispectral analysis confirms the assignment of 2DEV-DAS 1 to equilibration and provides definitive assignment of the peaks around 1708 cm^–1^ to the Red Chls.

The energy trapping component (2DES-DAS 4 and 2DEV-DAS 2) does not exhibit any pump wavelength dependence. All peaks decay toward the baseline synchronously on a ≈20 ps time scale, in agreement with the reported trapping lifetime for *Synechocystis sp*. PCC 6803.^33^ The loss of excitation dependence in the spectra is consistent with the 2D-DAS assignments, and with previous 2DEV studies on LHCII that have shown that the excitation dependence of the spectral features is lost upon completion of energy equilibration.^51^

To further test the assignment of the positive 13^1^-keto mode at 1708 cm^–1^ to the Red Chls we constructed a kinetic model to describe the equilibration and relaxation processes associated with the antenna Chls. From the kinetic model we generated model 2DES and 2DEV spectra, and then extracted the 2D-DAS to compare to the experimental 2D-DAS. Details are provided in Section S7 of the SI. The kinetic model that best reproduces the experimental 2D-DAS (Figure 3) is equilibration between the Bulk and Red Chls, followed by decay of the antenna excited states, where the positive bands in the 1708 cm^–1^ frequency region are assigned to the Red Chl states. We note that the kinetic model does not account for sub-ps components, such as those observed in the 2DES spectra; however, the model does qualitatively replicate the experimental 2DEV-DAS and 2DES-DAS with picosecond decay time constants, and hence further supports our assignment of the positive peaks at 1708 cm^–1^ to the Red Chls.

**Figure 3:**
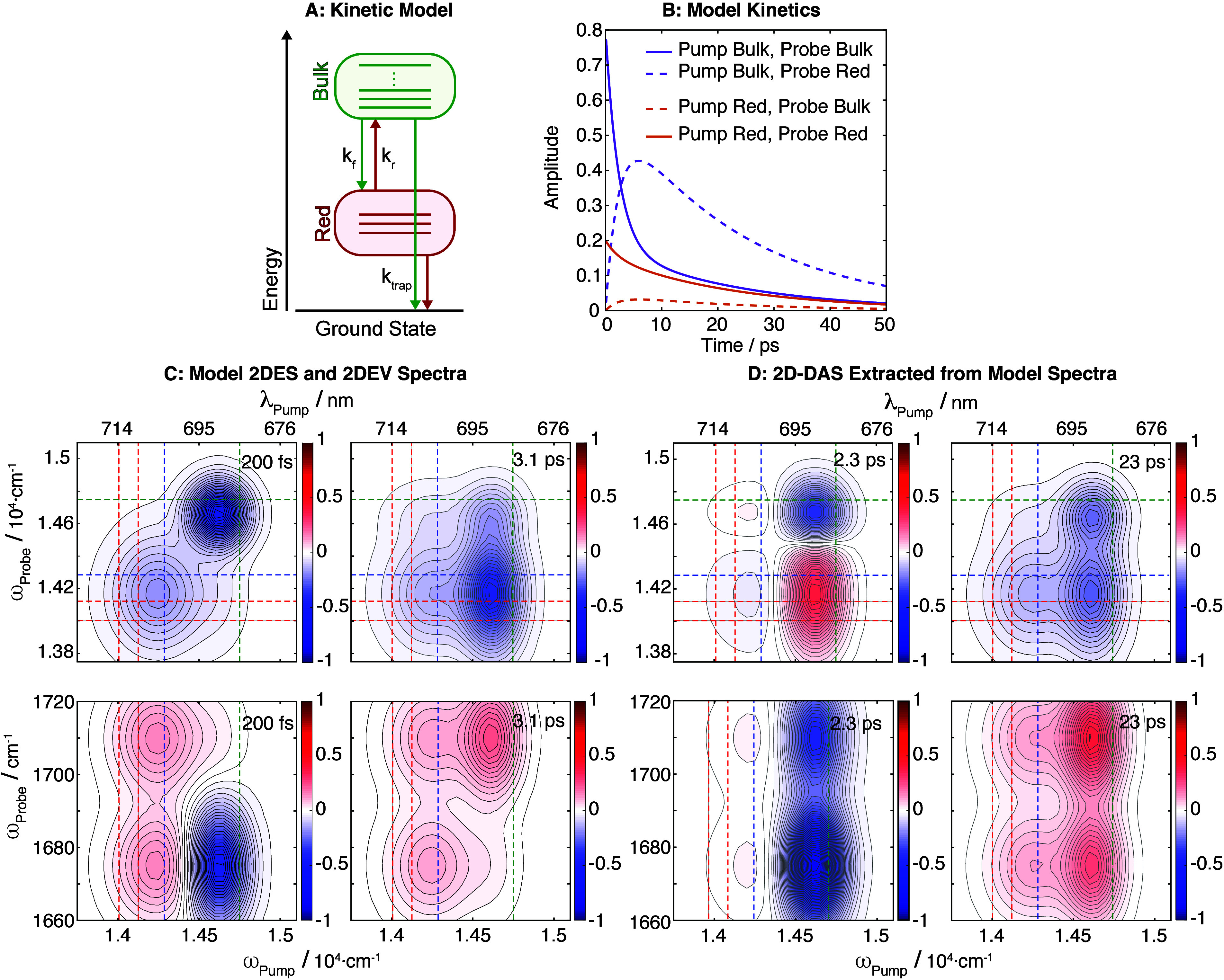
(A) Scheme illustrating the interconnectivity of the kinetic compartments representing the Bulk and Red Chls. (B) Concentration–time profiles obtained from solving the system of ordinary differential equations that correspond to the scheme in A. (C) Modeled multispectral 2D data obtained from the kinetic traces in B, which are used as the input to generate the 2D-DAS components shown in D.

Kinetic analysis has shown the excitation dependent temporal evolution of the 1708 cm^–1^ band is characteristic of a Red Chl excited state vibration. We also note that our measurements were performed on closed RCs, thus the 2DEV data cannot provide insight into the CS states of the RC. In the context of the previous experimental TRIR work,^40,41^ the 2DES and 2DEV spectra show that the cationic Chl features in this region of the mid-IR spectrum have contributions from the Red Chl excited states. This assignment is consistent with previous work and previously established models that assign the Red Chl excited states in PSI to mixed exciton-CT states.^16,21,25^ The local excited states of the Chls (Chl*Chl and ChlChl*), that comprise the Frenkel excitons, can couple with the interchlorophyll CT states (Chl^–^Chl^+^ and Chl^+^Chl^–^).^16,17^ The resulting excitonic states have some CT character due to the mixing. The 13^1^-keto vibrational mode associated with the 1708 cm^–1^ peak is assigned to the Chl^δ+^ component associated with the mixed FE/CT state.

As the peak at 1708 cm^–1^ corresponds to the Chl^δ+^ feature of the mixed exciton-CT state, one may also expect the Chl^δ−^ features to present in the spectra as well, where the vibrational frequency of the Chl^δ−^ keto mode is red-shifted compared to neutral Chl *a*.^40,57^ The vibrational absorption bands of anionic Chls in pigment–protein complexes have not been well-characterized, likely due to overlap with other spectral features. We suggest the Red Chl bands observed in the 1660–1690 cm^–1^ region could correspond to the C=O stretching modes of Chl^δ−^ associated with the exciton-CT states, which would be consistent with the observed kinetics and model spectra.

In this work, we have demonstrated the first application of 2DEV spectroscopy to cyanobacterial PSI in solution phase at room temperature as part of a multispectral 2D spectroscopic study. To interpret the spectra, we applied a 2D global analysis to extract the excited state kinetics of the photosynthetic antenna. The excitation dependent relaxation kinetics of the Bulk and Red Chls allowed for identification of the 13^1^-keto vibrations of the Red Chl mixed exciton-CT states, where the 1708 cm^–1^ band in the 2DEV spectra is assigned to the Chl^δ+^ component, and the photoinduced vibrational absorptions found at lower frequency (1660–1690 cm^–1^) are tentatively assigned to the anionic component, Chl^δ−^. By comparing the 2D-DAS extracted from the 2DES and 2DEV spectra, in addition to spectral modeling, we determined how energy transfer among the antenna Chls of PSI presents in the 2DEV spectra. The characterization of the initial Red Chl excited states as well as the analysis of the antenna kinetics detailed here will be critical for studying charge separation in PSI complexes with open reaction centers, where charge separation can take place. By comparing the 2DEV spectra of PSI with opened RCs to the data presented in this work with closed RCs, we will be able to better distinguish vibrational signals of the antenna Chls and RC Chls. This approach will allow for an effective separation of the energy transfer processes within the antenna chlorophylls from the electron transfer processes within the RC core. Thus, this study lays the groundwork for future studies of the electron transfer processes in PSI with 2DEV spectroscopy.

## Experimental Methods

PSI was isolated from *Synechocystis sp*. PCC 6803 using established procedures.^61^ A brief description is given in Section S8 of the SI. Experimental details for multispectral 2D experiments are provided in Sections S9 and S10 of the SI.

## Supplementary Material



## Data Availability

All data needed to evaluate the conclusions are provided in the paper and Supporting Information. The raw multispectral data are available from the corresponding author upon request.

## Notes

Details of the UV–vis and FTIR spectra of PSI, additional 2DES and 2DEV spectra, global fitting results and details on error propagation for 2D-DAS, information on the power dependent decay kinetics, details on preparation of the 2D spectral model, and experimental information on preparation of the PSI sample and the 2DEV spectrometer setup (PDF)
