# Supplementary material for: Isolating the Vibrational Spectra of the Red Chlorophylls in Photosystem I with Multispectral Two-Dimensional Spectroscopy
Source: J Phys Chem Lett. 2026 May 12;17(20):5718–26. doi: 10.1021/acs.jpclett.6c00658 (PMC13200252; doi:10.1021/acs.jpclett.6c00658)
Supplement: Supplementary file 1 [file jz6c00658_si_001.pdf]

Supporting Information

Isolating the Vibrational Spectra of the Red Chlorophylls in Photosystem I with Multispectral Two-Dimensional Spectroscopy

James D. Shipp,[a] Chenshuai Li,[b] Yumin Lee,[b] Michael Gorka,[c] John H. Golbeck,[d] and Jessica M. Anna[a,b]*

a) Department of Chemistry, University of Pittsburgh, Chevron Science Center, 219 Parkman Avenue, Pittsburgh, PA 15260, United States.

b) Department of Chemistry, University of Pennsylvania, 213 S. 34th Street, Philadelphia, PA, 19 104, United States

c) Department of Chemistry and Chemical Biology and The Baruch '60 Center for Biochemical Solar Energy Research, Rensselaer Polytechnic Institute, Troy, NY 12180, USA

d) Department of Biochemistry and Molecular Biology, The Pennsylvania State University, University Park, PA, 16802, United States.

Table of Contents

S1. UV-Vis and FTIR Spectra S3

S2. 2DES Spectra of Neutral and Oxidized Photosystem I S4

S3. 2DEV Spectra at Lower Detection Resolution S7

S4. Kinetic Trace Analysis S10

S5. Power Dependent Kinetics S11

S6. Global Fitting Procedure S13

S7. Modelling Multispectral 2D Data S15

S8. Preparation of PSI Samples S19

S9. Experimental Setup for 2DEV Spectroscopy S19

S10. Laser Pulse Compression Procedure S21

S11. References S23

S1. UV-Vis and FTIR Spectra

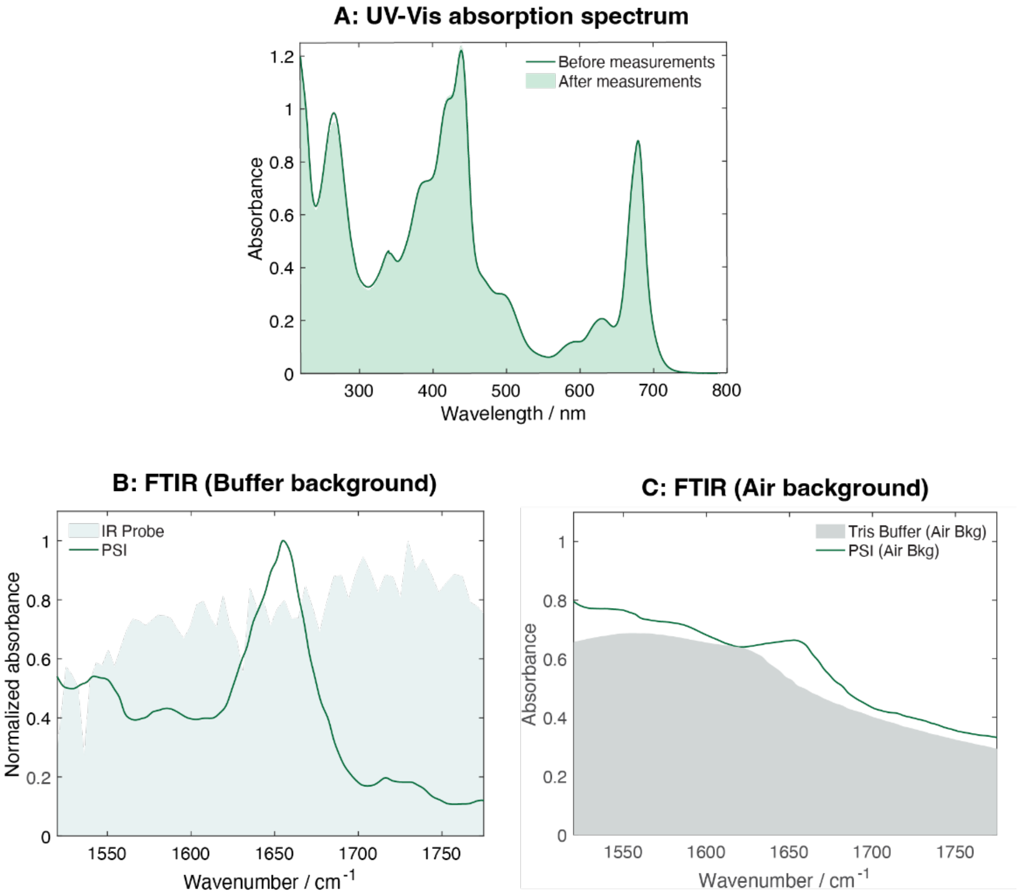

Figure S1. ***UV-Vis and FTIR Spectra of PSI.*** *Panel A plots the linear UV-Vis absorption spectrum of the PSI sample before (solid line) and after (shaded area) 2DEV measurements, demonstrating that no changes in the 550 – 750 nm region of the electronic spectrum occur due to sample decomposition during the measurement. B) Normalized FTIR spectrum of the PSI sample taken using the D_2O Tris buffer solution as a background in the 1520 – 1770 cm^{-1} region of the spectrum, overlaid with the spectrum of the IR probe pulse used for 2DEV measurements. C) FTIR spectra of the PSI sample and the D_2O Tris buffer with air as the background sample.*

S2. 2DES Spectra of Neutral and Oxidized Photosystem I

To show that we are working under conditions where P_{700} of PSI is photo-oxidized, we compare the 2DES spectra recorded under similar conditions to the 2DEV spectra, with a second sample that also included phenazine methosulfate (PMS) at a concentration of 60 mM to facilitate reduction of the photo-oxidized reaction center.[1,2] Further, the flow rate of the sample was increased from 2.5 cm^{-3} min^{-1} to 25 cm^{-3} min^{-1}. The addition of PMS and increase in sample flow rate resulted in observation of the long-lived charge separated state (CSS) by 2DES spectroscopy, as illustrated by the long-lived spectral features shown in Figure S2. The radical pair state is evidenced by the three peaks at detection frequencies of $1.4936x10^4$, $1.4443x10^4$, and $1.4155x10^4$ cm^{-1}, which correspond to wavelengths of 669, 692, and 706 nm. These are well characterized peaks of the cationic RC core.[3,4] In absence of PMS and at slow flow rates, the reaction center features are not observed due to photoaccumulation of $P_{700}^{\bullet+}$. Thus, we can consider the PSI sample as pre-oxidized in the 2DEV experiments. The absence of the CSS at slow flow rates and without PMS was further confirmed by global fitting of the 2DES spectra, where the characteristic positive peak at 692 nm was not detected. There are spectral features in the 'closed' (pre-oxidized) PSI sample along the diagonal axis of the spectrum at longer time delays. However, these are largely attributed to scattered pump light that is present across all time delays.

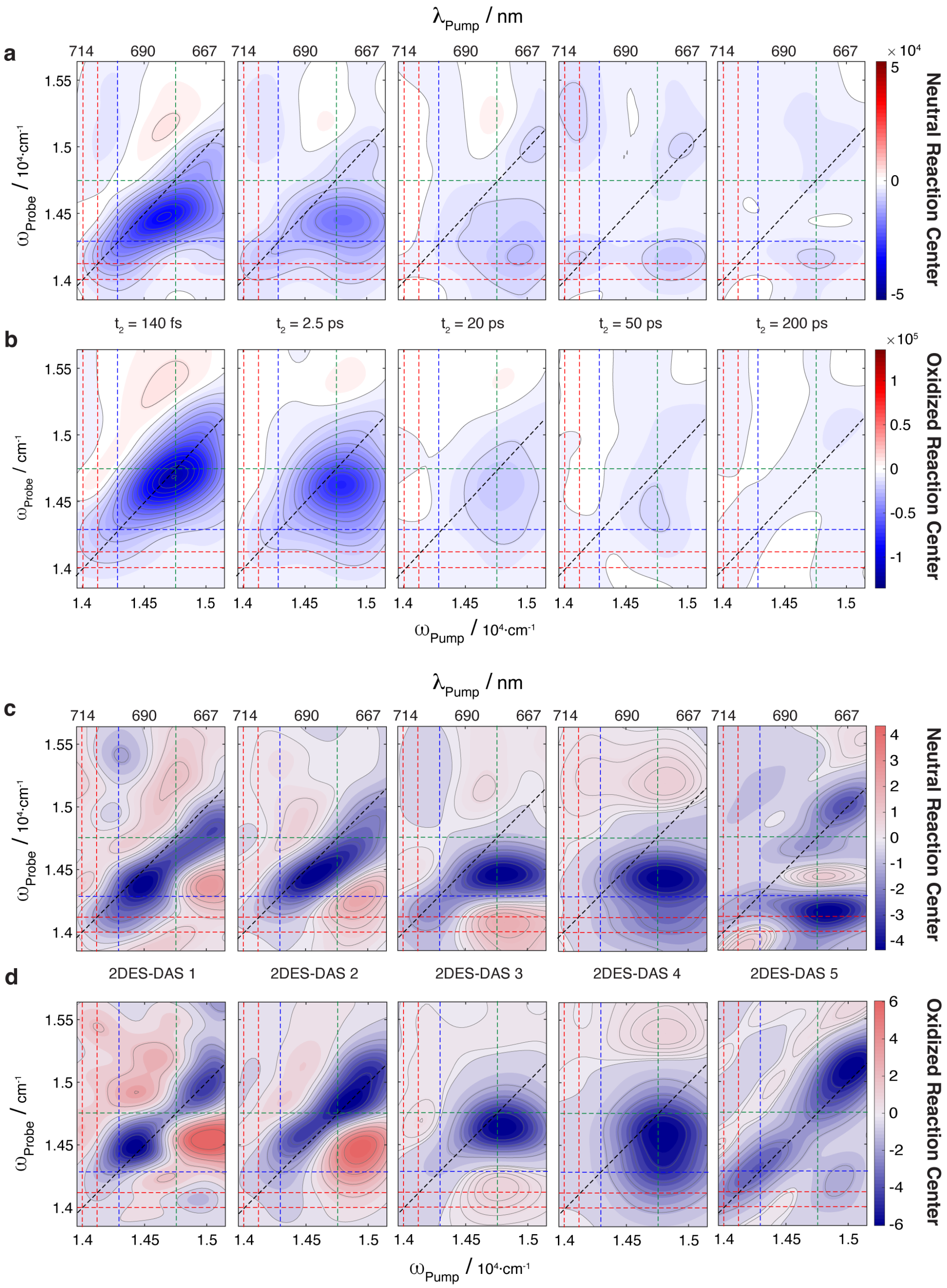

Figure S2. ***2DES and 2D-Decay Associated Spectra obtained for Neutral and Oxidized Photosystem I.*** *a,b) 2DES spectra obtained from PSI extracted from Synechocystis sp. PCC 6803 with a neutral (a) or pre-oxidized (b) P_{700} reaction center. Representative spectra are shown at t_2 time delays of 140 fs, 2.5 ps, 20 ps, 50 ps, and 200 ps. c,d) 2DES-DAS obtained by fitting of the 2DES spectra in a,b with five-components. The best fit decay constants are shown in Table S1.*

Table S1. *Fitted decay constants obtained from global lifetime analysis of the 2DES or 2DEV data, as well as the estimated error bars that represent the 95% confidence intervals. Averages of the decay constants extracted from the high- and low-resolution 2DEV data are also provided using the standard deviation of the two measurements as the error.*

**Kinetic Component**	**2DES (Neutral)**	**2DES (Pre-oxidized)**	**High Resolution 2DEV (Pre-oxidized)**	**Low Resolution 2DEV (Pre-oxidized)**	**Average 2DEV (Pre-oxidized)**
**t_1 / fs**	76 ± 1	94 ± 2	/	/	/
**t_2 / fs**	780 ± 10	710 ± 9.8	/	/	/
**t_3 / ps**	5.0 ± 0.06	3.9 ± 0.03	2.2 ± 0.05	3.0 ± 0.07	2.6 ± 0.57
**t_4 / ps**	23 ± 0.19	22 ± 0.13	22 ± 0.28	23 ± 0.32	22.5 ± 0.71
**t_5 / ns**	5000 (fixed)	5000 (fixed)	/	/	/

S3. 2DEV Spectra at Lower Detection Resolution

2DEV spectra were collected across a broader mid-IR spectral range through use of a lower resolution grating (50 gr mm^{-1} vs. 100 gr mm^{-1} for data in the main text). At lower resolution, the 2DEV detection axis spans the 1545 – 1800 cm^{-1} region of the mid-IR, and the data is shown in Figure S3. The spectral analysis discussed in the main text was repeated for the lower resolution data, where cross-sections along the detection axis and kinetic traces are shown in Figure S4 and S5, respectively. The analysis and interpretation of the peaks found in the lower resolution data in the 1660 – 1745 cm^{-1} region are consistent with those presented in the main text (Figure 2), where the 2DEV spectra in the main text are recorded at the higher detection axis resolution. The 13^1-keto vibrations of the charge neutral Chls are found in the 1610 – 1660 cm^{-1} region of the mid-IR spectrum. Consistent with previous studies,[5] we observe a strong positive feature centered at 1630 cm^{-1} and a shoulder at 1653 cm^{-1}, where the previous studies have assigned these transitions to the 13^1-keto vibrations of the charge neutral Chl excited states. In addition, we observe several weaker positive features superimposed on the 1630 cm^{-1} peak that have center frequencies of 1638, 1646, and 1661 cm^{-1}. To our knowledge definitive assignments for these spectral features are lacking.

The overlapped peaks in the 1610 – 1660 cm^{-1} region result in significant spectral congestion in the lower resolution 2DEV data. Therefore, in this work we do not focus on the analysis of the excited states of the charge neutral Chls.

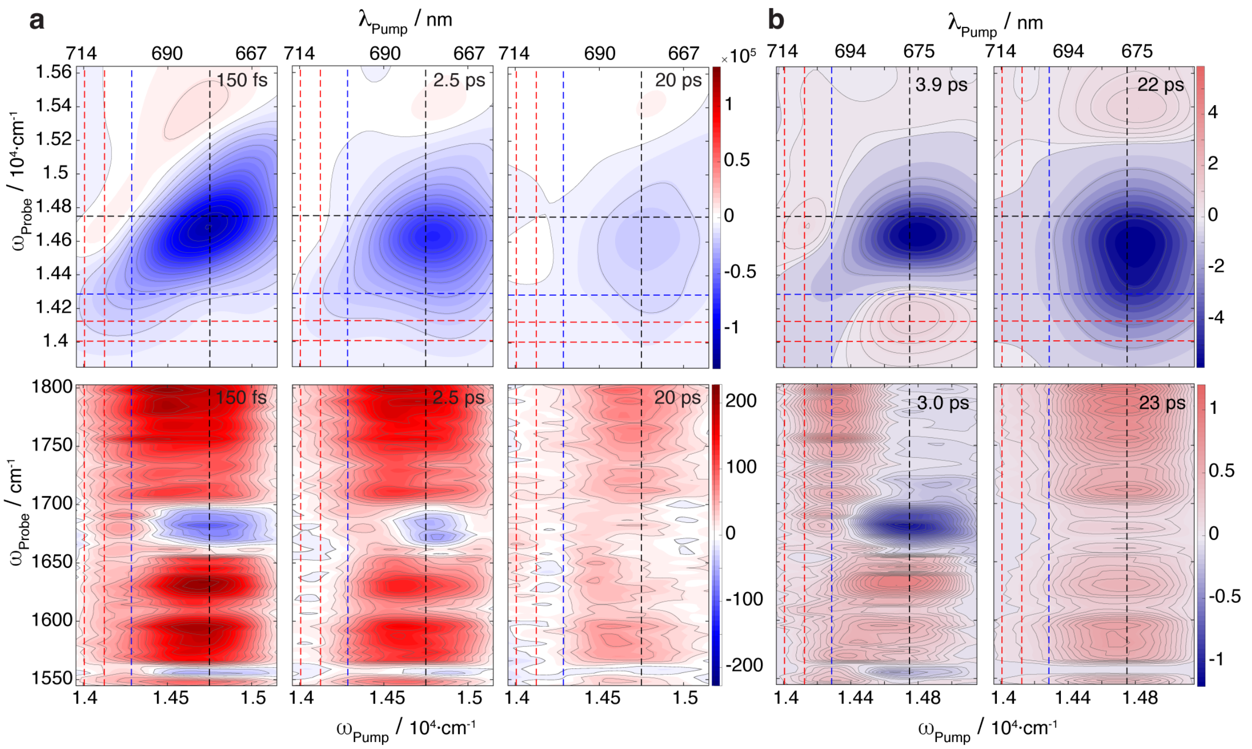

Figure S3. ***Multispectral 2D data of PSI extracted from Synechocystis sp. PCC 6803 and 2D-DAS obtained from independent global analyses of the 2DES and 2DEV data.*** *2DES and 2DEV spectra of pre-oxidized PSI extracted from Synechocystis sp. PCC 6803 in Tris buffer, collected following excitation with a visible pulse spanning 660 – 740 nm (1.35x10^4 – 1.52x10^4 cm^{-1}) and detection with a visible pulse spanning 620 – 746 nm (1.34x10^4 – 1.61x10^4 cm^{-1}) or a mid-IR pulse spanning 1545 – 1800 cm^{-1}. The IR probe was detected across a broader range at a lower resolution compared to the data shown in the main text. Correlation lines are drawn at pump wavelengths of 714, 708, 700, and 678 nm (1.400x10^4, 1.412x10^4, 1.43x10^4, and 1.475x10^4 cm^{-1}). For the spectra, positive amplitudes are shown in red and negative amplitudes are shown in blue. For the 2D-DAS, positive values (red) correspond to positive peak decay or negative peak growth and negative values (blue) correspond to positive peak growth or negative peak decay.*

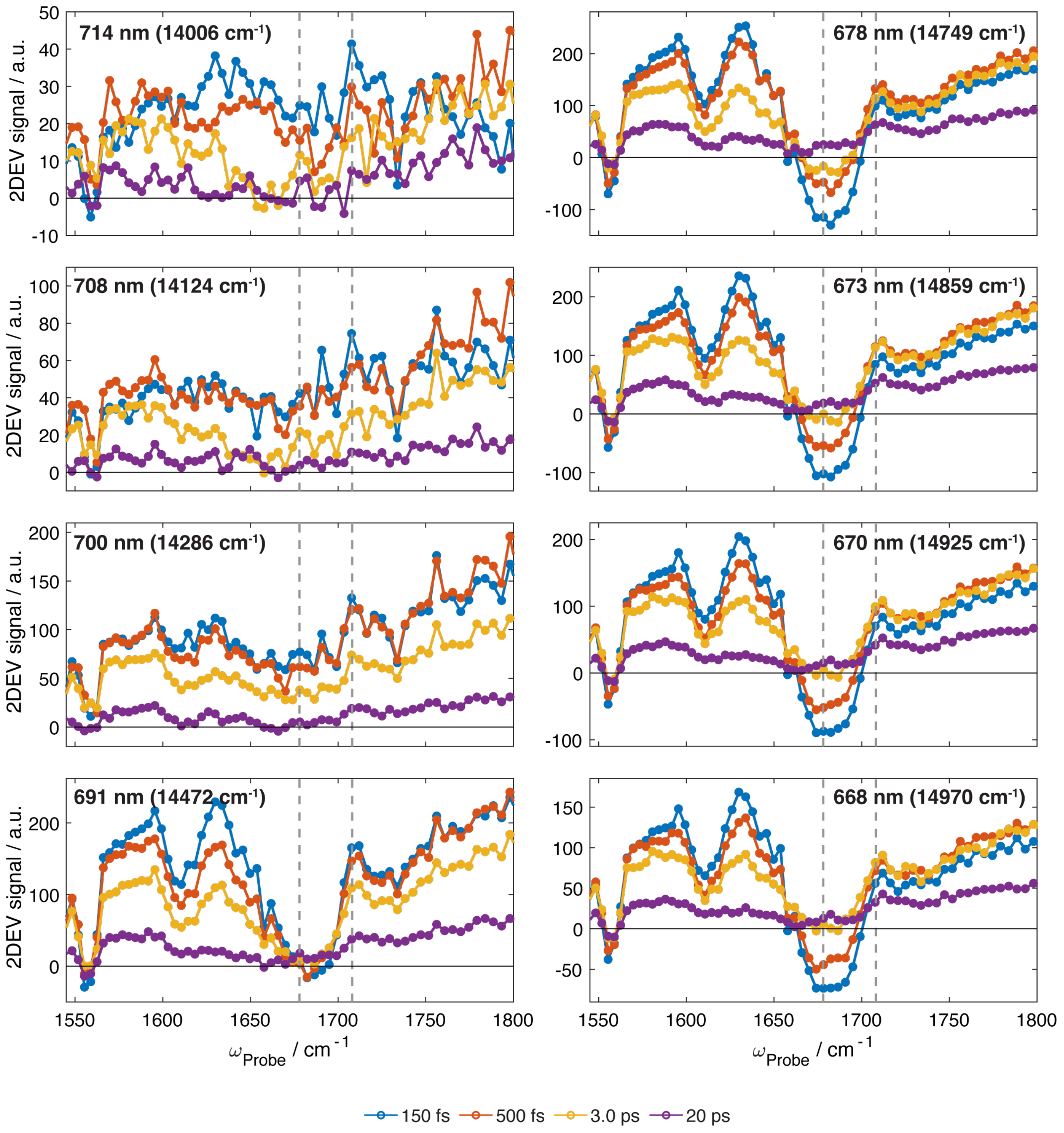

Figure S4. ***Cross-sections of the low resolution 2DEV spectra obtained at different visible excitation frequencies spanning the 1545 – 1800 cm^{-1} region along the mid-IR detection axis.*** *Slices taken along the probe axis are shown. The slices were taken from the 2DEV data shown in Figure S3. Correlation lines are drawn at 1678 and 1708 cm^{-1}, analogous to Fig. 2 in the main text. The excitation frequencies are shown on each graph.*

S4. Kinetic Trace Analysis

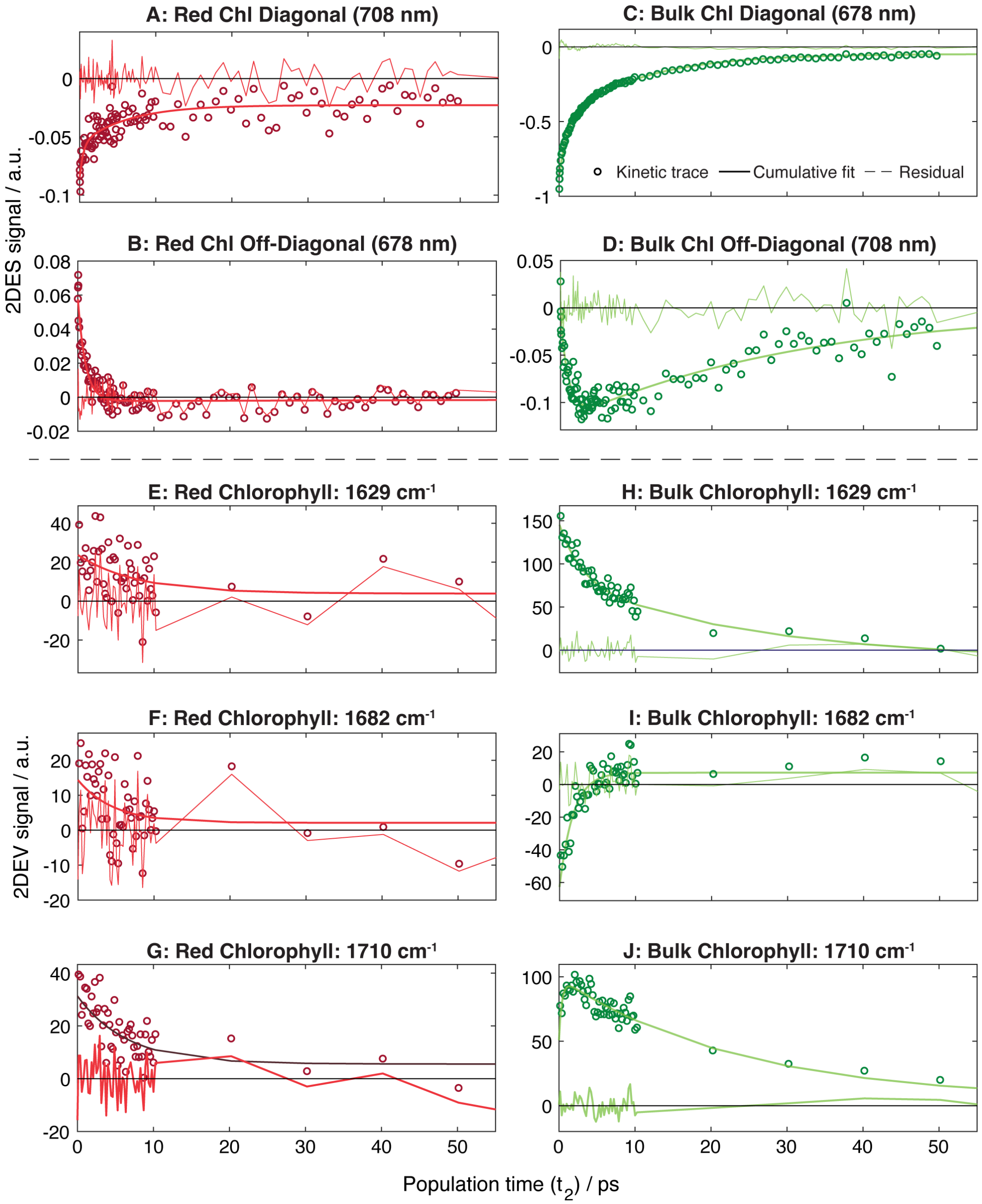

Figure S5. ***Time dependent kinetic traces obtained from the 2DES (A-D) and low resolution 2DEV (E-J) spectra.*** *The probe slices shown here were extracted from the 2DEV data shown in Figure S3. The kinetic traces (dots) correspond to the detection wavelengths/frequencies noted in the graph titles and are color coded for either Red Chl (red) or Bulk Chl (green) excitation. The thick solid lines represent best exponential fits of the kinetic data, and thin lines show the residual data after exponential fitting. Single phase exponential functions were used for peaks in the Red Chl excitation region of the 2DEV spectra. Biexponential functions were used for all 2DES kinetic traces and peaks in the Bulk Chl excitation region of the 2DEV spectra.*

S5. Power Dependent Kinetics

To determine the pump energy at which annihilation effects become significant due to multiple excitations of the PSI antenna, a power dependent series of transient absorption spectra (TA) were recorded (Figure S6), and the resulting data was exponentially fit to obtain the decay timescales of the antenna. TA was used for this purpose as the data collection time is much shorter compared to that of 2D spectra. Thus, a series of TA spectra could be recorded at pump powers of 4.5, 10, 15, 20, 25, and 32 nJ. The resulting spectra, kinetic traces at 678 nm, and time constants are provided in Figure S6.

The data were fit with a triexponential function, and the resulting time constants are plotted as a function of pump power. The first two decay constants, related to energy transfer within the PSI antenna, were insensitive to the excitation power. However, this was not the case for τ_3, where a linear decrease in the lifetime of the equilibrated antenna was observed. This decrease is a result of multi-photon excitation of the PSI antenna, and subsequent annihilation of the excited states.[3,6–8] The lowest sampled pump energy was 4.5 nJ, where annihilation effects were minimized. Thus, 5 nJ pump pulses were used for the multispectral experiments (where each pump pulse was 5 nJ).

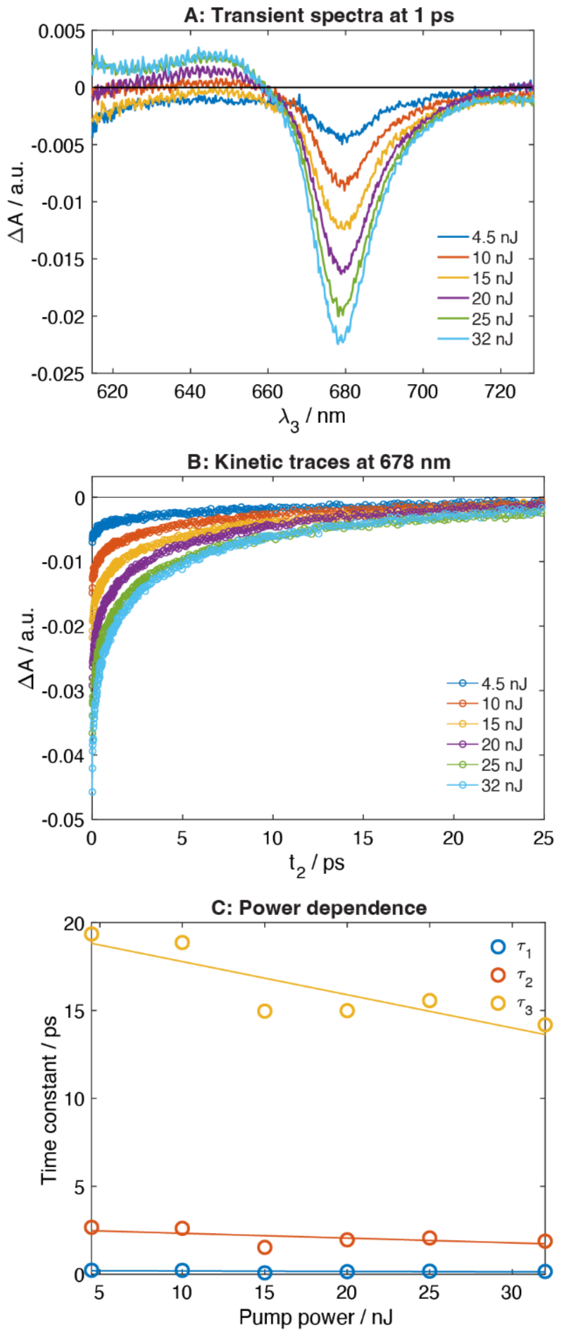

Figure S6. ***Power dependent lifetimes of the PSI antenna obtained by pump – probe transient absorption spectroscopy.*** *A) Power-dependent transient absorption spectra were obtained following electronic excitation of PSI with a visible pump pulse spanning 660 – 740 nm, the same range as those used for 2DES and 2DEV measurements, and representative measurements are displayed for a* t_2 *delay of 1 ps. B) Kinetic traces obtained from the maxima of the ground state bleach in the TA spectra. These kinetic traces are fit with a triexponential decay function and the output time constants are shown in C as a function of pump power, overlaid with linear fits of the relationship between pump power and the kinetic decay constants.*

S6. Global Fitting Procedure

Global lifetime analysis was carried out in MATLAB R2024. The 3D experimental data was reshaped to a 2D array such that the dimensions were N(λ_3 or ω_3) x [N(λ_1 or ω_1) x N(t_2)]. Global fitting was then carried out with the *lsqnonlin* function and Levenberg-Marquardt algorithm, as described previously.[9] The initial guess values of τ for 2DES-DAS fitting were 1/0.05, 1/0.23, 1/3.7, 1/16, and 1/5000 ps, with lifetime boundary conditions of [0, 0, 0, 0, 1/5000] and [Inf, Inf, Inf, Inf, 1/5000 ps]. For 2DEV-DAS, the initial guess values 1/3.7 and 1/16 ps, and lifetime boundary conditions of [0, 0] and [Inf, Inf] were used. After fitting, the resulting exponential amplitudes from each component were reshaped back to a 3D array matching the dimensions of the original 2D dataset (N(λ_3 or ω_3) x N(λ_1 or ω_1)) to retrieve the 2D-DAS.

To address the reduced number of components required to adequately describe the 2DEV data compared to the 2DES data, we can consider the temporal widths of the laser pulses used in the experiments. The pulse width of the visible probe was 15 fs, much shorter than the mid-IR probe (100 fs). This allows two additional sub-picosecond equilibration processes to be resolved from global analysis of the 2DES data, with decay timescales of 94 and 710 fs. These equilibration kinetics occur within the pulse overlap period of our 2DEV spectrometer, and are not resolved in the 2DEV spectra. Additionally, a fifth component was added to the 2DES model to account for residual signals that are present at the end of the experimental time delay which results from scattered pump light. This residual signal was modeled with a fixed decay time of 5 ns, which is effectively non-decaying on the experimental timescale. Such scattering is not observed in the 2DEV, as the pump scatter lies along the diagonal and the 2DEV measurements only monitor the cross-peak regions.

Error propagation for 2D-DAS decay constants

The error bars for the 2D-DAS decay constants report the 95% confidence intervals and are provided in Table S1. The error bars were obtained using MATLAB R2024 using a similar procedure to previous work in our group.[10] The *lsqnonlin* function was used to perform the required exponential fitting, from which the optimized rate constants, exponential amplitudes, residual data, and the Jacobian matrix were obtained. The variance (σ) of the residuals with the decay constants were obtained from Eq. S1. Here S_0 is the set of minimized values obtained from the non-linear fitting algorithm. N is the number of points in the input 2D data, and k is the number of fitting parameters, which we take as the number of time constants used for global fitting.

$$\sigma = \frac{S_0}{N - k} \qquad \text{Eq. S1}$$

From the Jacobian matrix (J) output by *lsqnonlin* we calculate a covariance matrix (Cov), as shown by Eq. S2, where the *–1* superscript corresponds to the matrix inverse and *T* corresponds to the matrix transpose.

$Cov = \sigma * (J^T * J)^{-1}$ Eq. S2

The standard errors for the optimized decay constants are obtained from Eq. S3. Where *diag* refers to matrix diagonalization.

$$\begin{pmatrix} \delta k_1 \\ \delta k_2 \\ \delta k_3 \\ \delta k_4 \\ \delta k_5 \end{pmatrix} = \sqrt{diag(Cov)}$$ Eq. S3

To obtain the 95% confidence interval we first determine the critical value (CV) for a twin-tailed probability distribution with the MATLAB *tinv* function, as shown by Eq. S4.

$CV = tinv(0.975, N - K)$ Eq. S4

With the critical value, we then obtain the upper and lower bounds of the decay constants and the 95% confidence interval (Eq S5 – S7). The analysis was performed on decay constants in units of ps^{-1}. These values are then converted to error bars in units of ps.

$k_{LB} = k_n - (CV * \delta k_n)$ Eq. S5

$k_{UB} = k_n + (CV * \delta k_n)$ Eq. S6

$k_n CI_{95\%} = k_{UB} - k_{LB}$ Eq. S7

S7. Modelling Multispectral 2D Data

Model spectra were generated to determine if the proposed kinetic model can qualitatively reproduce the observed spectral features in the 2DES and 2DEV datasets.

To produce the model spectra, we first write a set of differential rate equations based on the model, which are then analytically solved in MATLAB R2024a with the *dsolve* function. The resulting integrated rate equations are then evaluated with a set of input rate constants to obtain simulated concentration-time profiles for the kinetic mechanism. The rate constants for downhill (Bulk → Red) energy transfer, trapping of antenna states at the reaction center, and decay of the reaction center to the ground state were set to fixed values. The rate constant for uphill energy transfer (Red → Bulk) was set relative to the downhill transfer rate constant according to the Boltzmann relation (Eq. S8), where k_B is the Boltzmann constant (0.695 cm^{-1} K^{-1}), T is the temperature (K), ε_j-ε_i is the estimated energy difference between the Red Chl and the relaxed Bulk Chl states (cm^{-1}), and k_r and k_f are the rate constants of uphill and downhill energy transfer between the Bulk and Red Chls. The value of ε_j-ε_i was set to 250 cm^{-1}, which represents an estimate of the energy gap between the Red Chls and the relaxed excited states of the lower energy Bulk Chls. The forward and reverse rate constants were estimated from the average of the experimentally measured decay constant from the high and low resolution 2DEV-DAS 1 (k=0.3846 ps^{-1}), where we assumed that the averaged 2D-DAS decay constant represents the exchange rate constant associated with equilibration (k_{ex}). Using Eq. S8, assuming a temperature of 298 K, and using the fact that the exchange rate is the sum of k_f and k_r (k_{ex}= k_r + k_f.) we estimate k_f to be 0.2961 ps^{-1} (1/k_f = 3.378 ps) and k_r to be 0.0885 ps^{-1} (1/k_r= 11.29 ps).

$$k_r = k_f e^{-\left(\frac{\varepsilon_j - \varepsilon_i}{k_B T}\right)} \qquad \text{Eq. S8}$$

To create the model spectra from the simulated kinetic data we first generate a set of 2D Gaussian peaks for the 2DES and 2DEV spectra. The electronic center frequencies and linewidths were obtained from previously reported UV-Vis absorption spectra of PSI obtained at 77 K (Table S2).[11] The vibrational linewidths were set to a sigma value of 10 (23.5 cm^{-1}).

Table S2. *Absorption frequencies and FWHM values used in the model system for qualitative simulation of the multispectral data.*

Transition	Absorption Frequency / cm^{-1}	Absorption Wavelength / nm	FWHM / cm^{-1}
Bulk Chl: Q_y(0-1)	1.4661x10^4	682	471.3
Red Chl	1.4169x10^4	706	344.4

2DES Spectra

The model 2DES spectra were simulated by taking a similar approach to our previous work.[9] The spectra were produced using four 2D Gaussian peaks, which account for the two diagonal Red and Bulk Chl GSB/SEs and two Red and Bulk Chl off-diagonal GSB/SEs. The electronic linewidths along ω_{Pump} were set to be consistent with the identity of the excited Chls, the linewidths along ω_{Probe} were set to be consistent with the identity of the probed Chls. This gives the off-diagonal peaks an asymmetric lineshape in the model data. To model the evolution of these peaks over time, the 2D Gaussians were multiplied by the integrated rate equation of the corresponding Chl states, where the initial concentration of the pumped Chls is set to 1, and the initial concentration of the other Chls are set to 0.

2DEV Spectra

The model 2DEV spectra were generated in an analogous manner to the 2DES spectra. To produce model data that qualitatively reproduce the phenomena that we observe in the experimental data, model vibrational peaks were generated using four 2D Gaussian peaks. The 2D Gaussians mimic the vibrational features highlighted in the schematic spectra in Fig. 2A of the main text. The center frequencies and FWHM of the peaks along the ω_{Pump} axis were set to the values provided in Table S2. The position of the peaks along the ω_{Probe} axis was set as described below, where the peaks had a Gaussian FWHM value of 23.5 cm^{-1} (Gaussian $\sigma = 10$) along the IR detection axis.

The GSB feature (B_{V2}) found following Bulk Chls excitation was modeled by a negative peak at 1675 cm^{-1}, the kinetics of which were obtained from the Bulk Chl kinetic component of the integrated rate equations, analogous to peak B_E in the model 2DES spectra. The positive features identified following Red Chl excitation were represented by positive peaks at 1675 and 1710 cm^{-1}, modelling the features marked R_{V1} and R_{V2} in the main text. These peaks were set to follow the kinetics of the Red Chls obtained from the simulated kinetics, analogous to the R_E peak in the 2DES spectra. Negative peaks were not included in the model 2DEV spectra following Red Chl excitation, as these were not observed experimentally and we therefore do not know the relative amplitude of the positive and negative features at 1675 cm^{-1}.

The vibrational feature at 1710 cm^{-1} that is observed following Bulk Chl excitation (B_{V1}) was either assigned as a vibration of the Bulk or Red Chls in the model. If the peak was assigned to Bulk Chls the kinetics of this peak are analogous to peak B_E. If this feature was instead associated with the Red Chls, the peak followed the kinetics of the B_E' peak in the model 2DES spectra.

To approximate the effect of the spectral shape of the pump pulse spectrum on the 2DEV spectra, model 2DEV spectra were generated as described above and weighted by a Gaussian profile by multiplying the 2DEV spectra by a Gaussian with a center of 1.44×10^4 cm^{-1} and a width of 300 cm^{-1}, values which were obtained by fitting of the experimental pump pulse spectrum. The effect of weighting with the pump pulse is demonstrated in Figure S7, where simulated data are plotted

for the individual peaks associated with excitation of the Bulk Chls (Fig. S7A) and excitation of the Red Chls (Fig. S7B). When either the Bulk or Red Chls are excited, the corresponding peaks are centered at the frequencies noted in Table S2. Plotting the Bulk and Red Chl peaks together (the total simulated 2DEV spectra) (Fig. S7C) results in what appears to be a frequency shift. However, the transition frequencies were not changed, this is just due to the addition of the overlapping spectral features. The overlap also leads to what looks like a shoulder at ~690 nm excitation and 1710 cm^{-1} detection. Figure S7D plots the 2DEV spectra (Fig. S7C) weighted by the Gaussian profile, where we can think of the pump pulses as preferentially exciting different spectral features. Comparing Fig S7C to Fig. S7D we see that the pump pulses act to narrow the line shape and re-weight the amplitudes, where the transition frequencies are not altered, but the spectral components that have more overlap with the pump pulse are more intense.

A global analysis was performed on the simulated data and the resulting DAS were compared to the experimental 2DES-DAS and 2DEV-DAS shown in the main text.

The model that best reproduces the experimental results, for both 2DEV and 2DES, was equilibration between the Bulk and Red Chls, followed by irreversible trapping/quenching of energy at the reaction center, which causes the signal to decay. The positive vibrational peaks in the 1700 – 1720 cm^{-1} region were assigned to the Red Chl pools. This model uses two kinetic compartments, one for the Bulk Chls and one for the Red Chls. Electronic bands of the Bulk and Red Chls were produced using the center frequencies and FWHM values provided in Table S2. The reaction center is not explicitly included but serves as a trap, and the energy trapping is represented as a population decay. The kinetic model is summarized by the ordinary differential equations shown below (Eq. S10, S11).

$$\frac{d[A]}{dt} = -k_f[A]_t + k_r[B]_t - k_{trap}[A]_t \qquad \text{Eq. S10}$$

$$\frac{d[B]}{dt} = k_f[A]_t - k_r[B]_t - k_{trap}[B]_t \qquad \text{Eq. S11}$$

The terms in the ODEs are defined as follows:

- [A] = concentration of bulk chlorophylls
- [B] = concentration of red chlorophylls
- k_f = rate constant for downhill energy transfer from bulk to red chlorophylls
- k_r = rate constant for uphill energy transfer from red to bulk chlorophylls, set using the Boltzmann relationship.
- k_{trap} = rate constant for energy transfer from the antenna chlorophylls to the reaction center/trap

Table S3. Kinetic parameters used for the model multispectral 2D data

Parameter	Rate Constant / ps^{-1}
k_f	0.2961
k_r	0.0885
k_{trap}	0.043

Global analysis of the simulated data required two kinetic components, which were assigned to equilibration between Chl pools, and trapping at the RC core. The fit lifetimes were 2.6 and 23 ps. The 2D-DAS extracted from the simulated 2DES were able to qualitatively replicate the experimental 2D-DAS.

A second variant of the model was also tested where the IR absorption bands in the 1700 – 1720 cm^{-1} region were assigned to the Bulk Chls. This model 2DEV-DAS did not show any growth components above 1700 cm^{-1} following Bulk Chl excitation, thus this model cannot replicate the experimentally observed spectral features for the first 2DEV-DAS component.

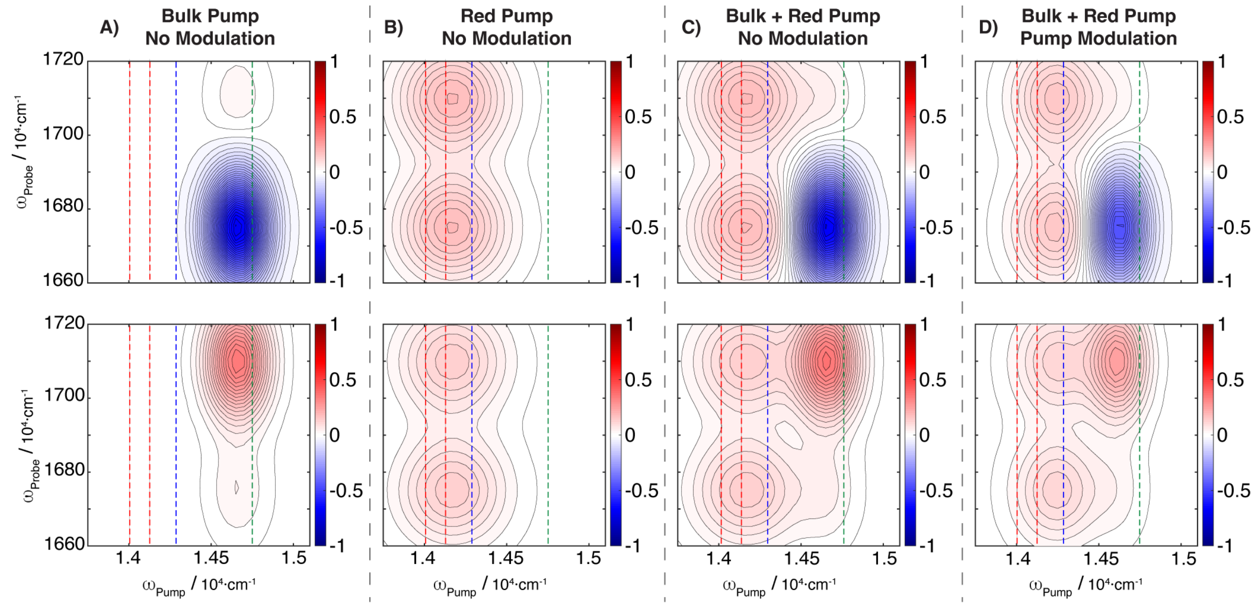

Figure S7. ***Modeled 2DEV Spectra.*** *Model spectra are generated as described in Section S7 for 2DEV spectra at t_2 = 200 fs (top) and t_2 = 3 ps (bottom). Spectral features ascribed to A) excitation of the Bulk Chls and B) excitation of the Red Chls without weighting by the pump pulse spectrum. C) Excitation of the Bulk and Red Chls (the total simulated 2DEV spectra) without weighting by the pump pulse. D) Excitation of the Bulk and Red Chls (the total simulated 2DEV) weighted by the pump pulse.*

S8. Preparation of PSI Samples

Thylakoid membranes were suspended at a concentration of 0.45 mg Chl cm^{-3} in 50 mM Tris HCl pH 8.3 containing 10 mM $CaCl_2$, and subsequently solubilized *via* incubation with 1% (w/v) n-dodecyl-β-maltoside (DDM) for 60 minutes in the dark at 4°C. Isolation of the solubilized PSI RC was accomplished by ultracentrifugation on a 5–20% sucrose gradient containing 0.05% DDM. The gradient was centrifuged at 4°C for 16 hours at 28,000 rpm in an SW-32 Ti rotor. After the first sucrose gradient, the trimeric band was removed, dialyzed to remove sucrose, and reapplied to a second 5–20% sucrose gradient to remove residual contaminants. Pure PSI was removed from the gradient and buffer-exchanged into 50 mM Tris HCl pH 8.3 containing 5% glycerol and stored at –80°C until used. D_2O exchange was accomplished by serial dilution/concentration over a 100 kDa centricon per an established procedure with slight modifications.[12] Purified PSI was diluted 20-fold with 25 mM Tris HCl pH 8.3, 0.05% DDM, 5% glycerol that was prepared in D_2O. The solution was allowed to incubate for at least 3 hours in the dark at 4°C before being concentrated over a 100 kDa Centricon that was pre-treated with D_2O. This process was repeated a total of 5 times. The final Chl concentration of the sample was ≈1.7 mg_{Chl} cm^{-3}. Samples for 2DEV were prepared in a demountable liquid cell (Harrick Scientific) equipped with 2 mm CaF_2 windows separated by a 50 μm Teflon spacer. The optical density of the sample was kept within the linear absorption regime for both visible and IR regions (0.9 at 680 nm, 0.1 at 1650 cm^{-1}).

S9. Experimental Setup for 2DEV Spectroscopy

2DEV spectroscopy was conducted as summarized below. A Ti: Sapphire source (Coherent Libra, 100 fs, 1 kHz) provided 800 nm pulses. 40% of the fundamental was used to generate 620 – 750 nm pulses with a home-built non-colinear optical parametric amplifier (NOPA). The pump was compressed with a Grism pair and an acousto-optic programmable dispersive filter (AOPDF) pulse shaper (Fastlite, Dazzler). The Gaussian time bandwidth of the pump was 15.8 fs, measured by SFG-FROG. The pump power was set to 5 nJ per pump pulse for all experiments. 60% of the fundamental was routed to a commercially available Optical Parametric Amplifier (TOPAS-C, Light Conversion), equipped with a difference frequency generation stage. A portion of the IR output was divided with a 50:50 beamsplitter to produce probe and reference pulses. Detection was achieved with a commercially available mercury-cadmium-telluride (MCT) detector (Infrared Systems Development), coupled with an automated spectrometer (HORIBA, iHR-320). The mid-IR portion of the optical table was purged with dry N_2 during the 2DEV experiments such that the relative humidity was below 5%. A four-frame phase cycling scheme was used to isolate the 2DEV signal. Data were referenced with the multichannel correlation method, as described previously.[13] The pump – probe polarization was set to be parallel for all measurements. For each t_2 delay, the t_1 delay was scanned from −0.1 – 80 fs in 0.312 fs steps, with a rotating frame of 350 THz. The sample was continuously translated in an oval pattern during measurements at a speed of 2.4 mm s^{-1} to reduce photodegradation. Sample decomposition was monitored between measurements by UV-Vis spectroscopy. To obtain 2DEV spectra with an improved signal:noise ratio for peak

identification, a total of 2000 averages of the t_1 scan were performed at each t_2 delay (-10, 0, 0.5, 3, 20 ps), where the total number of laser pulses was $2.048x10^6$ per 2DEV spectrum. For kinetic analysis, a longer t_2 vector was used (64 delays, −10 – 100 ps), with a total of 500 averages per t_2 delay ($5.12x10^5$ pulses). The averaging time was reduced to accommodate the liquid nitrogen depletion rate in the MCT detector (≈ 14 hours). The 2DEV experiments were repeated three times on different sample batches to ensure reproducibility of the results.

S10. Laser Pulse Compression Procedure

The visible pulses produced by the NOPA, shown in Figure 1 of the main text, were characterized by intensity auto- and cross-correlation with the sum-frequency generation frequency resolved optical gating (SFG-FROG) method. The pump and probe pulses were compressed in two stages:

i) Probe autocorrelation, compressed with a double-pass grating compressor and four-pass prism compressor[14] and characterized by SHG-FROG, using a 0.05 mm BBO crystal (t_{FWHM} = 20.5 fs, Pulse width = 14.5 fs).

ii) Pump/probe cross-correlation, where the pump was compressed with a double pass Grism compressor and AOPDF pulse shaper (Fastlite, Dazzler) and characterized by SFG-FROG, using a 0.05 mm BBO crystal (t_{FWHM} = 22.3 fs, Pulse width = 15.8 fs).

The IR probe pulse produced by the OPA / DFG had a temporal FWHM bandwidth of approximately 100 fs.[10,15] Therefore, delays shorter than 150 fs were not included in any data analysis.

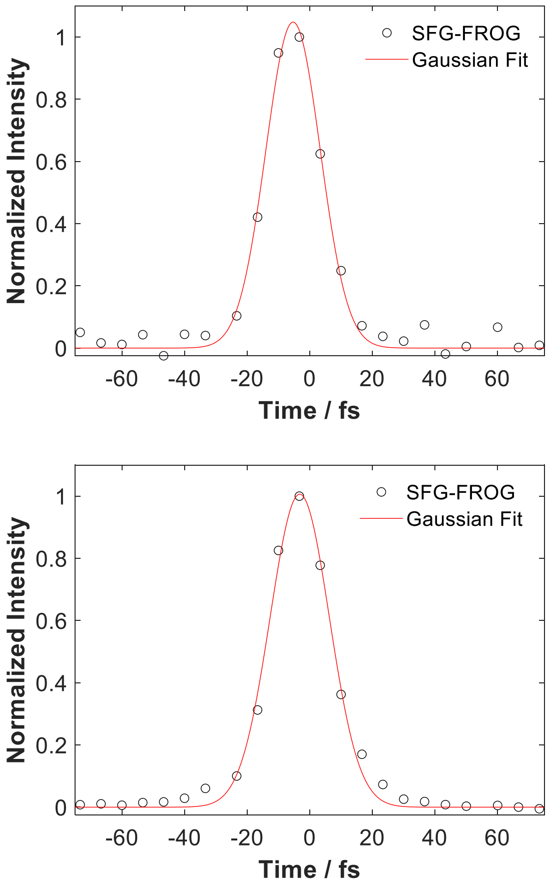

Figure S8. ***Projections of the SFG-FROG signals obtained from the compressed visible pulses.*** *top) Projected SFG-FROG for the broadband visible probe pulses, where the Gaussian pulse width obtained from fitting was 14.5 fs. bottom) Projected SFG-FROG for the broadband visible pump pulses, where the Gaussian pulse width obtained from fitting was 15.8 fs.*

S11. References

(1) Byrdin, M.; Rimke, I.; Schlodder, E.; Stehlik, D.; Roelofs, T. A. Decay Kinetics and Quantum Yields of Fluorescence in Photosystem I from Synechococcus Elongatus with P_{700} in the Reduced and Oxidized State: Are the Kinetics of Excited State Decay Trap-Limited or Transfer-Limited? *Biophys. J.* **2000**, *79* (2), 992–1007. https://doi.org/10.1016/S0006-3495(00)76353-3.

(2) Giera, W.; Ramesh, V. M.; Webber, A. N.; van Stokkum, I.; van Grondelle, R.; Gibasiewicz, K. Effect of the P_{700} Pre-Oxidation and Point Mutations near A_0 on the Reversibility of the Primary Charge Separation in Photosystem I from Chlamydomonas Reinhardtii. *Biochim. Biophys. Acta - Bioenerg.* **2010**, *1797* (1), 106–112. https://doi.org/10.1016/j.bbabio.2009.09.006.

(3) Savikhin, S.; Xu, W.; Chitnis, P. R.; Struve, W. S. Ultrafast Primary Processes in PSI from Synechocystis Sp. PCC 6803: Roles of P_{700} and A_0. *Biophys. J.* **2000**, *79* (3), 1573–1586. https://doi.org/10.1016/S0006-3495(00)76408-3.

(4) Herascu, N.; Hunter, M. S.; Shafiei, G.; Najafi, M.; Johnson, T. W.; Fromme, P.; Zazubovich, V. Spectral Hole Burning in Cyanobacterial Photosystem I with P_{700} in Oxidized and Neutral States. *J. Phys. Chem. B* **2016**, *120* (40), 10483–10495. https://doi.org/10.1021/acs.jpcb.6b07803.

(5) Zamzam, N.; Kaucikas, M.; Nürnberg, D. J.; Rutherford, A. W.; van Thor, J. J. Femtosecond Infrared Spectroscopy of Chlorophyll f-Containing Photosystem I. *Phys. Chem. Chem. Phys.* **2019**, *21* (3), 1224–1234. https://doi.org/10.1039/C8CP05627G.

(6) Causgrove, T. P.; Yang, S.; Struve, W. S. Polarized Pump-Probe Spectroscopy of Photosystem I Antenna Excitation Transport. *J. Phys. Chem.* **1989**, *93* (18), 6844–6850. https://doi.org/10.1021/j100355a053.

(7) Gillie, J. K.; Small, G. J.; Golbeck, J. H. Nonphotochemical Hole Burning of the Native Antenna Complex of Photosystem I (PSI-200). *J. Phys. Chem.* **1989**, *93* (4), 1620–1627. https://doi.org/10.1021/j100341a085.

(8) Lyle, P. A.; Struve, W. S. Temperature Dependence of Antenna Excitation Transport in Native Photosystem I Particles. *J. Phys. Chem.* **1991**, *95* (10), 4152–4158. https://doi.org/10.1021/j100163a047.

(9) Lee, Y.; Gorka, M.; Golbeck, J. H.; Anna, J. M. Ultrafast Energy Transfer Involving the Red Chlorophylls of Cyanobacterial Photosystem I Probed through Two-Dimensional Electronic Spectroscopy. *J. Am. Chem. Soc.* **2018**, *140* (37), 11631–11638. https://doi.org/10.1021/jacs.8b04593.

(10) Weng, W.; Weberg, A. B.; Gera, R.; Tomson, N. C.; Anna, J. M. Probing Ligand Effects on the Ultrafast Dynamics of Copper Complexes via Midinfrared Pump-Probe and 2DIR Spectroscopies. *J. Phys. Chem. B* **2021**, *125* (44), 12228–12241. https://doi.org/10.1021/acs.jpcb.1c06370.

(11) Tros, M.; Mascoli, V.; Shen, G.; Ho, M.-Y.; Bersanini, L.; Gisriel, C. J.; Bryant, D. A.; Croce, R. Breaking the Red Limit: Efficient Trapping of Long-Wavelength Excitations in Chlorophyll-f-Containing Photosystem I. *Chem* **2021**, *7* (1), 155–173. https://doi.org/10.1016/j.chempr.2020.10.024.

(12) Kim, S.; Sacksteder, C. A.; Bixby, K. A.; Barry, B. A. A Reaction-Induced FT-IR Study of Cyanobacterial Photosystem I. *Biochemistry* **2001**, *40* (50), 15384–15395. https://doi.org/10.1021/bi0110241.

(13) Feng, Y.; Vinogradov, I.; Ge, N.-H. General Noise Suppression Scheme with Reference

Detection in Heterodyne Nonlinear Spectroscopy. *Opt. Express* **2017**, *25* (21), 26262. https://doi.org/10.1364/oe.25.026262.
(14) Akturk, S.; Gu, X.; Kimmel, M.; Trebino, R. Extremely Simple Single-Prism Ultrashort-Pulse Compressor. *Opt. Express* **2006**, *14* (21), 10101. https://doi.org/10.1364/OE.14.010101.
(15) Askelson, P. G.; Meloni, S. L.; Hoffnagle, A. M.; Anna, J. M. Resolving the Impact of Hydrogen Bonding on the Phylloquinone Cofactor through Two-Dimensional Infrared Spectroscopy. *J. Phys. Chem. B* **2022**, *126* (48), 10120–10135. https://doi.org/10.1021/acs.jpcb.2c03556.
